# Supplementary material for: A Likelihood Approach for Real-Time Calibration of Stochastic Compartmental Epidemic Models
Source: PLoS Comput Biol. 2017 Jan 17;13(1):e1005257. doi: 10.1371/journal.pcbi.1005257 (PMC5240920; doi:10.1371/journal.pcbi.1005257)
Supplement: S1 File — (TAR.GZ) [file pcbi.1005257.s014.tar.gz › HSPH_Online-SI-Revision/EnKF07/n10-mild/n10-mild_EnKF2_table-8.pdf]

1	newly 3 weeks spec	108.	154.69	{28., 418.}	116.694	0.385108	newly 3 weeks cumul	274.	799.42	{258., 1732.}	198.606	0.300229	newly 5 weeks spec	177.	58.59	{3., 171.}	84.661	0.958749	newly 5 weeks cumul	569.	951.95	{315., 2363.}	110.047	0.262923	newly 58 weeks cumul	3351.	1064.01	{338., 3128.}	80.3059	0.778971
2	newly 3 weeks spec	133.	369.08	{0., 817.}	250.105	0.944011	newly 3 weeks cumul	288.	3803.6	{731., 9716.}	1223.29	0.961336	newly 5 weeks spec	307.	118.97	{0., 350.}	86.798	1.61464	newly 5 weeks cumul	831.	4123.29	{765., 10 788.}	403.277	0.576771	newly 63 weeks cumul	4314.	4356.42	{777., 11 775.}	67.2582	0.350692
3	newly 3 weeks spec	119.	858.21	{83., 1724.}	637.084	0.851212	newly 3 weeks cumul	252.	4290.6	{1385., 9207.}	1605.63	1.13646	newly 5 weeks spec	232.	468.75	{1., 1079.}	150.168	0.72858	newly 5 weeks cumul	651.	5385.36	{2120., 10 568.}	731.315	0.862897	newly 66 weeks cumul	4852.	6774.47	{2444., 11 249.}	62.9305	0.246486
4	newly 3 weeks spec	134.	23.41	{0., 60.}	83.9179	1.52631	newly 3 weeks cumul	346.	601.19	{180., 1065.}	105.199	0.300568	newly 5 weeks spec	273.	6.1	{0., 13.}	97.7656	2.50269	newly 5 weeks cumul	805.	618.02	{192., 1193.}	53.8981	0.319727	newly 61 weeks cumul	4621.	626.5	{194., 1277.}	86.4423	0.994493
5	newly 3 weeks spec	76.	122.16	{0., 182.}	210.447	1.37455	newly 3 weeks cumul	221.	714.3	{112., 536.}	282.262	0.333677	newly 5 weeks spec	121.	75.52	{0., 136.}	133.835	2.22465	newly 5 weeks cumul	442.	884.99	{129., 857.}	208.613	0.53364	newly 41 weeks cumul	2815.	1139.57	{131., 2364.}	102.628	1.13081
6	newly 3 weeks spec	89.	956.92	{31., 2038.}	1000.94	0.947564	newly 3 weeks cumul	165.	4459.95	{1071., 8667.}	2608.76	1.33299	newly 5 weeks spec	215.	507.69	{0., 1139.}	198.256	1.04768	newly 5 weeks cumul	499.	5674.73	{1250., 9710.}	1046.23	1.01475	newly 67 weeks cumul	3503.	6792.12	{1364., 10 740.}	120.686	0.441804
7	newly 3 weeks spec	131.	262.52	{0., 753.}	195.115	1.24177	newly 3 weeks cumul	306.	3730.12	{714., 10 657.}	1123.57	0.912468	newly 5 weeks spec	265.	77.2	{0., 264.}	85.7358	1.87639	newly 5 weeks cumul	790.	3943.1	{730., 10 777.}	407.709	0.573304	newly 63 weeks cumul	3676.	4109.84	{730., 11 829.}	74.3194	0.356876
8	newly 3 weeks spec	120.	150.26	{19., 377.}	113.683	0.504793	newly 3 weeks cumul	269.	795.23	{248., 1564.}	208.978	0.38178	newly 5 weeks spec	198.	60.96	{2., 155.}	85.8182	1.09065	newly 5 weeks cumul	652.	950.11	{304., 1971.}	109.71	0.395785	newly 58 weeks cumul	4528.	1095.58	{320., 3519.}	81.0592	0.982433
9	newly 3 weeks spec	122.	99.39	{17., 154.}	62.2869	0.374721	newly 3 weeks cumul	305.	2100.4	{938., 6068.}	591.77	0.726892	newly 5 weeks spec	202.	16.21	{0., 27.}	94.7871	1.97543	newly 5 weeks cumul	662.	2154.26	{949., 6074.}	230.018	0.420719	newly 62 weeks cumul	4588.	2168.79	{949., 6074.}	62.3093	0.493292
10	newly 3 weeks spec	146.	182.69	{2., 770.}	156.062	0.933058	newly 3 weeks cumul	372.	1065.83	{148., 3407.}	236.605	0.412604	newly 5 weeks spec	191.	77.76	{0., 74.}	110.377	1.808	newly 5 weeks cumul	713.	1251.45	{166., 4152.}	168.658	0.544842	newly 59 weeks cumul	3268.	1500.13	{171., 7263.}	92.3602	0.955602
11	newly 3 weeks spec	101.	577.52	{96., 1625.}	482.139	0.646519	newly 3 weeks cumul	225.	4061.3	{767., 9354.}	1708.45	1.10062	newly 5 weeks spec	200.	154.45	{5., 358.}	75.045	0.627082	newly 5 weeks cumul	597.	4502.04	{980., 10 526.}	662.362	0.777845	newly 64 weeks cumul	2927.	4748.04	{1063., 10 733.}	93.7643	0.324371
12	newly 3 weeks spec	49.	932.21	{41., 2147.}	1816.35	1.17323	newly 3 weeks cumul	71.	4761.36	{711., 9706.}	6610.23	1.66079	newly 5 weeks spec	277.	471.65	{1., 1041.}	119.895	0.691975	newly 5 weeks cumul	466.	5890.64	{1221., 11 271.}	1174.3	1.04933	newly 66 weeks cumul	3893.	7368.25	{2789., 12 732.}	103.493	0.361801
13	newly 3 weeks spec	133.	128.37	{23., 373.}	91.2556	0.500867	newly 3 weeks cumul	290.	713.71	{252., 1438.}	161.486	0.358804	newly 5 weeks spec	295.	52.94	{3., 144.}	87.3424	1.23624	newly 5 weeks cumul	831.	847.5	{312., 1969.}	84.13	0.412443	newly 58 weeks cumul	4651.	950.78	{330., 2765.}	80.6381	0.955679
14	newly 3 weeks spec	178.	213.18	{3., 794.}	155.36	0.972966	newly 3 weeks cumul	407.	1477.1	{178., 6865.}	315.012	0.40517	newly 5 weeks spec	248.	36.28	{0., 50.}	95.4435	1.87698	newly 5 weeks cumul	867.	1597.64	{213., 7258.}	186.002	0.55554	newly 59 weeks cumul	4541.	1635.2	{224., 7299.}	87.5816	0.990067
15	newly 3 weeks spec	89.	27.97	{0., 66.}	79.6517	1.22621	newly 3 weeks cumul	247.	527.74	{145., 1034.}	137.725	0.346556	newly 5 weeks spec	149.	9.24	{0., 12.}	94.2282	2.14516	newly 5 weeks cumul	522.	551.64	{150., 1109.}	58.5479	0.285895	newly 61 weeks cumul	4384.	567.61	{158., 1109.}	87.0527	1.02407
16	newly 3 weeks spec	118.	154.91	{19., 174.}	105.653	0.37337	newly 3 weeks cumul	242.	2328.46	{767., 6412.}	865.413	0.817663	newly 5 weeks spec	266.	36.05	{0., 46.}	95.8308	1.82221	newly 5 weeks cumul	698.	2435.62	{900., 6428.}	257.246	0.422342	newly 62 weeks cumul	4382.	2491.02	{929., 6429.}	67.9868	0.507208
17	newly 3 weeks spec	32.	189.8	{3., 858.}	550.438	0.619282	newly 3 weeks cumul	75.	1275.8	{150., 6617.}	1610.43	0.779443	newly 5 weeks spec	45.	31.63	{0., 53.}	105.578	1.11234	newly 5 weeks cumul	154.	1377.66	{166., 6748.}	804.26	0.541205	newly 59 weeks cumul	3428.	1413.67	{172., 6748.}	95.2681	1.04225
18	newly 3 weeks spec	96.	213.95	{2., 801.}	242.406	0.902611	newly 3 weeks cumul	238.	1371.49	{171., 6335.}	494.769	0.426246	newly 5 weeks spec	159.	39.47	{0., 57.}	97.7547	1.84417	newly 5 weeks cumul	518.	1502.8	{177., 6483.}	259.031	0.468559	newly 59 weeks cumul	2823.	1571.15	{182., 7212.}	98.9639	0.895026
19	newly 3 weeks spec	250.	163.22	{19., 374.}	79.44	0.553154	newly 3 weeks cumul	538.	2345.19	{904., 6345.}	339.608	0.510264	newly 5 weeks spec	410.	31.03	{0., 89.}	92.4317	1.99946	newly 5 weeks cumul	1241.	2446.06	{921., 6359.}	118.422	0.273732	newly 62 weeks cumul	4288.	2476.82	{922., 6360.}	62.6334	0.454053
20	newly 3 weeks spec	215.	297.18	{0., 783.}	134.679	1.20857	newly 3 weeks cumul	419.	3636.87	{714., 9013.}	769.797	0.780584	newly 5 weeks spec	303.	99.29	{0., 308.}	89.3234	1.83707	newly 5 weeks cumul	946.	3896.75	{735., 10 707.}	323.439	0.518548	newly 63 weeks cumul	4612.	4108.04	{740., 11 739.}	64.2142	0.365791
21	newly 3 weeks spec	171.	280.52	{0., 768.}	157.345	1.14519	newly 3 weeks cumul	333.	3622.45	{732., 8942.}	988.766	0.88424	newly 5 weeks spec	270.	81.12	{0., 266.}	91.0667	1.8553	newly 5 weeks cumul	859.	3846.45	{756., 10 541.}	355.279	0.541779	newly 63 weeks cumul	4235.	4013.13	{756., 11 545.}	62.8484	0.331078
22	newly 3 weeks spec	162.	265.21	{2., 899.}	195.5	1.02044	newly 3 weeks cumul	330.	1679.96	{162., 7663.}	447.618	0.433189	newly 5 weeks spec	235.	54.62	{0., 77.}	97.7106	1.85587	newly 5 weeks cumul	771.	1853.16	{174., 7888.}	234.638	0.568989	newly 59 weeks cumul	3786.	1930.55	{178., 7896.}	94.2026	0.943746
23	newly 3 weeks spec	145.	579.32	{100., 1006.}	312.786	0.535196	newly 3 weeks cumul	243.	3843.09	{1175., 9351.}	1485.58	1.0696	newly 5 weeks spec	283.	158.81	{10., 356.}	64.5901	0.636999	newly 5 weeks cumul	754.	4292.48	{1326., 10 536.}	476.531	0.674302	newly 64 weeks cumul	3569.	4536.64	{1369., 10 778.}	66.2118	0.277826
24	newly 3 weeks spec	208.	255.68	{0., 555.}	166.798	1.20176	newly 3 weeks cumul	535.	696.72	{91., 1624.}	130.71	0.564743	newly 5 weeks spec	227.	109.18	{0., 476.}	112.211	1.81445	newly 5 weeks cumul	960.	986.79	{91., 2359.}	126.178	0.723779	newly 60 weeks cumul	2818.	1173.02	{97., 3615.}	93.6842	1.04363
25	newly 3 weeks spec	8.	873.21	{40., 1743.}	10 818.1	1.76316	newly 3 weeks cumul	23.	4734.3	{1077., 9497.}	20 483.9	2.18163	newly 5 weeks spec	113.	458.85	{0., 1036.}	363.69	1.11159	newly 5 weeks cumul	209.	5832.27	{1299., 9661.}	2691.31	1.34111	newly 67 weeks cumul	3078.	6923.03	{1508., 10 446.}	143.533	0.402258
26	newly 3 weeks spec	173.	126.23	{6., 337.}	90.0751	0.64442	newly 3 weeks cumul	463.	744.72	{32., 1382.}	112.795	0.368614	newly 5 weeks spec	234.	48.8	{2., 128.}	85.6325	1.20565	newly 5 weeks cumul	924.	869.02	{39., 1868.}	92.6645	0.514962	newly 58 weeks cumul	3097.	965.39	{44., 2558.}	81.9926	0.877963
27	newly 3 weeks spec	144.	350.62	{1., 986.}	271.931	1.15285	newly 3 weeks cumul	310.	873.19	{8., 2490.}	254.487	0.613775	newly 5 weeks spec	330.	159.	{0., 857.}	109.042	1.96076	newly 5 weeks cumul	867.	1297.29	{8., 4279.}	167.097	0.835984	newly 60 weeks cumul	4073.	1527.11	{8., 7328.}	89.5357	1.24561
28	newly 3 weeks spec	96.	365.96	{4., 1092.}	381.333	0.912782	newly 3 weeks cumul	277.	1403.84	{52., 4495.}	439.978	0.518841	newly 5 weeks spec	113.	161.62	{0., 641.}	175.487	1.56305	newly 5 weeks cumul	500.	1814.	{74., 6778.}	330.96	0.5922	newly 38 weeks cumul	2478.	2202.99	{100., 8615.}	110.826	0.828998
29	newly 3 weeks spec	215.	29.5	{0., 65.}	87.507	1.61395	newly 3 weeks cumul	511.	539.7	{149., 1106.}	60.7397	0.297353	newly 5 weeks spec	312.	8.11	{0., 14.}	97.4006	2.49385	newly 5 weeks cumul	1054.	561.86	{162., 1309.}	59.7704	0.454421	newly 61 weeks cumul	4420.	573.61	{163., 1309.}	87.0224	1.02674
30	newly 3 weeks spec	272.	205.36	{2., 290.}	131.662	1.21067	newly 3 weeks cumul	607.	674.58	{18., 2197.}	126.886	0.689325	newly 5 weeks spec	287.	77.23	{0., 192.}	100.422	2.14995	newly 5 weeks cumul	1151.	892.94	{19., 2265.}	113.487	0.860304	newly 60 weeks cumul	4309.	990.94	{20., 2302.}	89.6537	1.28627
31	newly 3 weeks spec	58.	954.52	{32., 1964.}	1563.41	1.05038	newly 3 weeks cumul	93.	4468.68	{1086., 8715.}	4707.38	1.54535	newly 5 weeks spec	156.	505.89	{0., 1202.}	280.583	1.04348	newly 5 weeks cumul	351.	5690.88	{1266., 9530.}	1526.96	1.12096	newly 67 weeks cumul	3019.	6811.58	{1388., 10 710.}	148.655	0.440163
32	newly 3 weeks spec	113.	107.61	{0., 460.}	142.239	1.6068	newly 3 weeks cumul	325.	1171.44	{75., 3722.}	357.428	0.644817	newly 5 weeks spec	137.	10.54	{0., 30.}	94.365	2.47659	newly 5 weeks cumul	581.	1210.99	{78., 3850.}	227.726	0.771787	newly 40 weeks cumul	3452.	1218.57	{78., 3856.}	91.0078	1.24534
33	newly 3 weeks spec	152.	28.77	{0., 66.}	84.0066	1.42323	newly 3 weeks cumul	297.	576.68	{156., 1141.}	126.815	0.343792	newly 5 weeks spec	297.	7.31	{0., 14.}	97.5387	2.5063	newly 5 weeks cumul	807.	596.82	{167., 1263.}	58.0967	0.372391	newly 61 weeks cumul	4655.	605.63	{169., 1263.}	86.9897	1.04948
34	newly 3 weeks spec	162.	29.13	{0., 73.}	84.8951	1.51889	newly 3 weeks cumul	346.	583.97	{154., 1316.}	103.864	0.330093	newly 5 weeks spec	262.	7.84	{0., 14.}	97.0076	2.51286	newly 5 weeks cumul	775.	605.92	{162., 1341.}	52.5935	0.332782	newly 61 weeks cumul	4348.	618.87	{165., 1361.}	85.7666	0.983911
35	newly 3 weeks spec	234.	25.04	{0., 52.}	94.5897	1.91876	newly 3 weeks cumul	519.	741.55	{153., 1358.}	92.0674	0.361431	newly 5 weeks spec	339.	4.	{0., 8.}	98.8201	2.85428	newly 5 weeks cumul	1155.	755.1	{159., 1358.}	64.0139	0.475479	newly 61 weeks cumul	4450.	758.58	{160., 1358.}	83.6306	0.986447
36	newly 3 weeks spec	156.	349.42	{0., 801.}	203.718	1.03623	newly 3 weeks cumul	432.	3928.09	{752., 9846.}	812.905	0.817056	newly 5 weeks spec	336.	99.81	{0., 285.}	87.7887	1.75004	newly 5 weeks cumul	978.	4205.29	{775., 10 751.}	339.829	0.537632	newly 63 weeks cumul	4342.	4376.39	{781., 11 759.}	66.3918	0.343283
37	newly 3 weeks spec	100.	947.01	{36., 2147.}	866.89	0.918728	newly 3 weeks cumul	185.	4745.29	{769., 9686.}	2473.56	1.28273	newly 5 weeks spec	184.	480.27	{3., 1048.}	201.571	0.728677	newly 5 weeks cumul	548.	5896.55	{1380., 11 172.}	986.582	0.976784	newly 66 weeks cumul	3080.	7378.14	{2447., 12 445.}	151.951	0.428786
38	newly 3 weeks spec	166.	148.87	{20., 172.}	89.6325	0.464261	newly 3 weeks cumul	393.	2449.35	{909., 6434.}	529.148	0.648085	newly 5 weeks spec	256.	38.65	{0., 35.}	96.9492	1.95995	newly 5 weeks cumul	843.	2560.49	{924., 6444.}	213.152	0.372339	newly 62 weeks cumul	4770.	2620.03	{935., 6444.}	67.0291	0.516328
39	newly 3 weeks spec	121.	265.37	{0., 754.}	206.653	1.11211	newly 3 weeks cumul	299.	3610.59	{731., 8983.}	1108.34	0.92433	newly 5 weeks spec	257.	78.72	{0., 277.}	91.7276	1.83647	newly 5 weeks cumul	736.	3823.12	{759., 10 696.}	423.584	0.584421	newly 63 weeks cumul	3570.	3983.12	{760., 11 750.}	66.6913	0.304953
40	newly 3 weeks spec	88.	228.02	{12., 572.}	220.773	0.503611	newly 3 weeks cumul	286.	1371.61	{603., 3498.}	387.584	0.540729	newly 5 weeks spec	121.	77.78	{0., 133.}	98.0661	1.14823	newly 5 weeks cumul	519.	1582.78	{617., 3975.}	213.461	0.341419	newly 36 weeks cumul	2280.	1691.19	{617., 4109.}	83.611	0.484991
41	newly 3 weeks spec	60.	948.51	{38., 1949.}	1498.65	1.07911	newly 3 weeks cumul	169.	4251.68	{845., 8648.}	2420.32	1.2843	newly 5 weeks spec	165.	541.74	{0., 1260.}	283.503	1.02368	newly 5 weeks cumul	429.	5529.18	{1074., 9234.}	1196.16	1.0368	newly 67 weeks cumul	3396.	6713.28	{1212., 10 691.}	127.663	0.446091
42	newly 3 weeks spec	170.	242.15	{3., 818.}	175.512	0.999822	newly 3 weeks cumul	363.	1637.67	{176., 7611.}	394.344	0.446553	newly 5 weeks spec	211.	51.75	{0., 63.}	98.8009	1.90376	newly 5 weeks cumul	769.	1796.51	{209., 7848.}	226.025	0.566339	newly 59 weeks cumul	3469.	1878.28	{218., 7850.}	96.1712	0.91966
43	newly 3 weeks spec	65.	643.88	{29., 1313.}	907.938	0.826783	newly 3 weeks cumul	145.	5779.56	{1644., 9956.}	3888.59	1.50964	newly 5 weeks spec	321.	159.04	{0., 426.}	83.2523	1.2169	newly 5 weeks cumul	595.	6250.34	{1709., 10 667.}	955.382	0.963093	newly 65 weeks cumul	3665.	6438.65	{1863., 11 638.}	94.5149	0.311677
44	newly 3 weeks spec	156.	27.53	{0., 66.}	85.4423	1.44354	newly 3 weeks cumul	333.	542.73	{155., 1088.}	99.2763	0.320081	newly 5 weeks spec	232.	8.26	{0., 14.}	96.4397	2.37005	newly 5 weeks cumul	768.	565.04	{165., 1308.}	51.9948	0.339818	newly 61 weeks cumul	4274.	579.55	{167., 1312.}	86.4401	0.999533
45	newly 3 weeks spec	48.	456.85	{19., 1098.}	871.521	0.819254	newly 3 weeks cumul	71.	3839.18	{458., 9153.}	5311.1	1.47221	newly 5 weeks spec	143.	296.39	{0., 1023.}	218.105	1.3365	newly 5 weeks cumul	285.	4488.14	{561., 9174.}	1481.92	1.02149	newly 68 weeks cumul	4420.	5539.21	{595., 9773.}	81.4215	0.436494
46	newly 3 weeks spec	89.	579.6	{19., 1134.}	595.753	0.792649	newly 3 weeks cumul	211.	5962.43	{1576., 10 992.}	2734.18	1.36049	newly 5 weeks spec	199.	151.12	{0., 427.}	94.7739	1.30235	newly 5 weeks cumul	585.	6399.69	{1576., 12 167.}	1005.17	1.00017	newly 65 weeks cumul	3593.	6600.43	{1576., 12 345.}	109.681	0.416432
47	newly 3 weeks spec	73.	744.12	{32., 1715.}	940.247	0.873731	newly 3 weeks cumul	110.	5899.19	{1806., 10 139.}	5266.3	1.62161	newly 5 weeks spec	151.	203.6	{0., 573.}	114.543	0.920963	newly 5 weeks cumul	352.	6484.53	{2050., 11 486.}	1747.75	1.18996	newly 65 weeks cumul	4475.	6772.91	{2557., 12 448.}	75.8474	0.294174
48	newly 3 weeks spec	120.	151.15	{19., 180.}	95.575	0.364462	newly 3 weeks cumul	221.	2307.8	{693., 6427.}	946.932	0.864817	newly 5 weeks spec	260.	33.99	{0., 46.}	96.85	1.85503	newly 5 weeks cumul	652.	2409.81	{837., 6440.}	275.244	0.441006	newly 62 weeks cumul	3589.	2462.41	{931., 6440.}	67.715	0.436443
49	newly 3 weeks spec	81.	118.03	{9., 277.}	113.148	0.438688	newly 3 weeks cumul	266.	691.39	{31., 1148.}	184.515	0.395515	newly 5 weeks spec	116.	43.97	{1., 124.}	84.3707	0.996821	newly 5 weeks cumul	474.	803.97	{40., 1518.}	115.437	0.361717	newly 58 weeks cumul	3702.	894.9	{46., 1992.}	83.174	0.97602
50	newly 3 weeks spec	137.	102.92	{4., 177.}	83.8394	0.600709	newly 3 weeks cumul	367.	619.91	{25., 743.}	114.15	0.388237	newly 5 weeks spec	235.	40.1	{2., 86.}	87.3277	1.25337	newly 5 weeks cumul	808.	722.89	{30., 969.}	84.0854	0.509335	newly 58 weeks cumul	2666.	837.56	{39., 1229.}	83.5499	0.89582
